# Assessing whether EORTC QLQ-30 and FACT-G measure the same constructs of quality of life in patients with total laryngectomy

**DOI:** 10.1186/s12955-018-1012-x

**Published:** 2018-09-14

**Authors:** Kamyar Iravani, Peyman Jafari, Allahkaram Akhlaghi, Bijan Khademi

**Affiliations:** 1 Department of Otorhinolaryngology, Shiraz University of Medical Sciences, Shiraz, Iran; 2 Department of Biostatistics, Shiraz University of Medical Sciences, Shiraz, Iran

**Keywords:** Quality of life, Total laryngectomy, EORTC QLQ-C30, FACT-G

## Abstract

**Background:**

The European Organization for Research and Treatment of Cancer QOL Core Questionnaire 30 (EORTC QLQ-30) and the Assessment of Cancer Therapy-General (FACT-G) are the two most widely used measures of cancer-specific health-related quality of life (HRQOL). This study aims to assess whether the two instruments measure the same constructs of HRQOL in patients with total laryngectomy.

**Methods:**

The EORTC QLQ-30 and the FACT-G was completed by 132 patients with total laryngectomy. Convergent, discriminant, and construct validity of the EORTC QLQ-C30 and the FACT-G were assessed by Spearman’s correlation and explanatory factor analysis.

**Results:**

The results of factor analysis showed that the EORTC QLQ-C30 and the FACT-G measure different aspects of HRQOL. Moreover, both instruments showed excellent convergent and discriminant validity, except for nausea and vomiting symptom subscale in the EORTC QLQ-C30 questionnaire. The internal consistency was close or greater than 0.7 for all domains of both instruments except for functional wellbeing in FACT-G.

**Conclusions:**

This study revealed that neither of the two instruments can be replaced by the other in the assessment of HRQOL in Iranian patients with total laryngectomy. Accordingly, clinicians should exactly define their research questions related to patient-reported outcomes before choosing which instrument to use.

## Background

Total laryngectomy is still considered the primary method of treatment for those diagnosed with advanced laryngeal cancer [1]. Laryngeal cancer predominantly affects men, with a male to female ratio of 7:1 to 10:1. In men, laryngeal cancer comprises 2.4% of all cancers and 2.1% of all cancer deaths worldwide [2, 3]. In addition to total laryngectomy, there are other treatment modalities for laryngeal cancer including partial laryngectomy, transoral laser microsurgery (TLM), and combined chemoradiation alone or after surgery. Despite advances in treatment options and survival of patients after treatment, health-related quality of life (HRQOL) remains a major issue in patients with total laryngectomy [2, 4]. Voice problems, swallowing difficulties, pain, tracheostomy problems and taste disorders all have negative impacts on the (QOL) after treatment of laryngeal cancer. Psychological distress, depression, anxiety, and communication problems have been found to be common among laryngeal cancer patients after diagnosis and treatment of the disease.

Although there are numerous instruments available to measure the concepts related to HRQOL, choosing the most appropriate QOL measure is the first objective in clinical research. The Treatment of Cancer Quality of Life Questionnaire Core 30 (EORTC QLQ-C30) and the Functional Assessment of Cancer Therapy—General (FACT-G) are the most frequently used questionnaires to measure HRQOL in patients with cancer. Both questionnaires are generic, multidimensional and originally designed to assess QOL in patients with all types of cancer and are not specifically introduced for head and neck cancer. In general, the EORTC QLQ-C30 restricts its items to relatively objective aspects of functioning, whereas the FACT-G encourages patients to reflect on their thoughts and feelings throughout [5]. Although psychometric properties of the EORTC QLQ-C30 [6–15] and FACT-G [16–27] have been previously evaluated in different languages and clinical settings, psychometric evidence is not decisive in recommending one instrument or the other. Recently, a systematic review was set out to compare the EORTC QLQ-C30 and FACT-G HRQOL instruments with an aim to informing choice between them [5]. This literature review showed that there are differences between the social domains, scale structure and tone that inform choice for any particular study. Although a number of studies have simultaneously compared EORTC QLQ-C30 and FACT-G instruments using the same sample, such an explanation has never been provided in patients with total laryngectomy. Accordingly, this study is designed to evaluate whether the EORTC QLQ-C30 and FACT-G HRQOL measure the same constructs of HRQOL in Iranian patients with total laryngectomy. The second goal of the present research is to test whether one instrument has superior internal consistency and validity compared with the other. This study assists researchers to decide which of the two QOL instruments is more suitable in routine clinical practice.

## Methods

### Participants and instruments

This is a cross-sectional study which was carried out among 132 Iranian patients with total laryngectomy who were treated at the Khalil Hospital affiliated to the Shiraz University of Medical Sciences, Shiraz, Iran, from 2015 to 2016. All patients completed two self-administered HRQOL questionnaires including EORTC QLQ-C30 and FACT-G. Participants signed the informed consent forms, and they were instructed in detail how to fill out the instruments. The demographic data, including age and gender were also collected. The EORTC QLQ-C30 is a 30-item core cancer generic questionnaire for measuring HRQOL in cancer patients. The Persian version of the EORTC QLQ-C30 was previously evaluated and approved by the EORTC Study Group on QOL [9]. The EORTC QLQ-C30 contains five functional subscales including physical functioning (five items), role functioning (two items), emotional functioning (four items), cognitive functioning (two items), social functioning (two items), nine symptom subscales, a global health status, and one isolated item. Nine symptom scales or items in the EORTC QLQ-C30 include three-item symptom scale measuring fatigue, two-item symptom scales measuring pain and nausea and vomiting, and six single-item symptom scales measuring dyspnoea, insomnia, appetite loss, constipation, diarrhoea and financial impact.

All participants responded to the items of the functional subscales on a four-point Likert scale ranging from “not at all” to “very much”. The raw subscale scores were transformed into a 0–100 scale in which 0 indicating poor QOL and 100 excellent. The FACT-G is comprised of four subscales: physical wellbeing (seven items), social/family well-being (seven items), emotional wellbeing (six items), and functional wellbeing (seven items). All questions in the FACT-G use a five-point rating scale (0 = not at all, 1 = a little bit, 2 = somewhat, 3 = quite a bit, and 4 = very much). All subscales in the FACT-G had a score range from 0 to 28, except for the wellbeing domain, which had a score range from 0 to 26.

### Statistical analysis

The internal contingency of the QOL subscales was evaluated using Cronbach’s alpha coefficient. Internal contingency was considered satisfactory if the coefficient was greater than 0.7. Convergent and discriminant validity was assessed using Spearman’s correlation. The value of a correlation coefficient of greater than 0.40 between an item and its own hypothesized scale provides evidence of convergent validity. Discriminant validity is supported whenever a correlation between an item and its hypothesized scale is higher than its correlation with the other scales. A scaling success is counted if the item-to-own scale correlation is significantly higher than the correlations of the item-to-other scale [28]. Exploratory factor analysis with an iterated principal factor approach and varimax rotation for the nine separate subscales (five from the EORTC QLQ-30 and four from the FACT-G), were used to test whether the two instruments measure the same construct of HRQOL. Analysis of variance was also used to compare HRQOL across taste and also among different types of post-surgery radiation. Likewise, we examined the range and distribution of responses to each item including ceiling and floor effects that occur when responses on a questionnaire cluster at the more negative or positive health state. The presence of ceiling or floor effects in certain items indicates that they have poor discrimination which may lead to reduce precision and responsiveness of the questionnaire. In general, a total of less than 40% respondents selecting “not at all” or “very much” indicates that an item does not show substantial floor or ceiling effects [29]. All analyses were conducted using SPSS, version 16.0.

## Results

The characteristics of the participants included in the study are listed in Table 1. The results of convergent and discriminant validity, and also internal consistency, of the Persian version of the FACT-G and EORTC QLQ-C30 for patients with laryngeal tumor are presented in Table 2. The Cronbach’s alpha coefficients (internal consistency) are close or greater than 0.7 in all domains of the functional scales of the EORTC QLQ-C30. However, for symptom scales of the EORTC QLQ-C30 questionnaire, alpha coefficients were less than 0.7. Moreover, the alpha coefficients are greater than 0.7 in all domains of the FACT-G except for the functional wellbeing subscale. The results show that both questionnaires have good convergent and discriminant validity. The scaling success rates for convergent and discriminant validity are equal or close to 100% in all subscales of FACT-G and EORTC QLQ-C30 except for nausea and vomiting in the symptom scale of the EORTC QLQ-C30. The exploratory factor analysis with varimax rotation was used to determine whether the FACT-G and functional scales of the EORTC QLQ-C30 measure the same constructs of HRQOL or not. As shown in Table 3, the first factor includes all domains of EORTC QLQ-C30, and the second factor extracted encompasses all domains of the FACT-G except the social functioning domain, which had a weak correlation with their own domain. The results of comparing health QOL scores across taste and post-surgery radiation subgroups are shown in Table 4. Accordingly, there was no significant difference between patients’ (QOL) in none of the two variables. Moreover, the findings revealed that in the FACT-G, all items had floor and ceiling effects less than 19% and 38%, respectively. While in the EORTC QLQ-C30 all items had floor effects less than 12%, 16 items had ceiling effects greater than 40%.

**Table 1:** Characteristics of study participants

Characteristics	Number/value	Percentage
Gender		
Male	98	74.2
Female	43	25.8
Age		
Mean (SD)	62.65(7.18)	–
Post-surgery radiation		
Radio therapy	11	8.40
Chemotherapy	18	13.6
Both of them	46	34.8
None of them	57	43.2
Taste		
Increase	6	4.60
Decrease	63	47.7
Without change	63	47.7
Stage		
III	86	65.1
IV	46	34.8
Time after surgery (month)		
Mean (SD)	2.62(0.33)	–

**Table 2:** Internal consistency and item scaling test including convergent and discriminant validity for the FACT-G and the EORTC QLQ-C30 subscales

Scale	No.items	α	Mean ± SD	Convergent validity		discriminant validity	
				Range of correlation	Scaling success (percent)	Range of correlation	Scaling success (percent)
FACT-G							
Physical wellbeing	7	0.81	17.21 ± 5.42	0.51–0.84	7/7(100)	0.002–0.49	21/21(100)
Social/family wellbeing	7	0.75	17.16 ± 5.15	0.41–0.79	7/7(100)	0.05–0.37	19/21(90)
Emotional wellbeing	6	0.72	13.03 ± 4.79	0.19–0.81	5/6(83)	0.02–0.39	15/18(83)
Functional wellbeing	7	0.61	14.73 ± 3.98	0.44–0.62	7/7(100)	0.01–0.27	21/21(100)
EORTC QLQ-C30							
Global health status/QOL	2	0.84	53.66 ± 22.35	0.92–0.94	2/2(100)	0.16–0.60	28/28(100)
Functional scales							
Physical functioning	5	0.69	75.25 ± 17.35	0.50–0.75	5/5(100)	0.03–0.62	62/70(88)
Role functioning	2	0.83	76.26 ± 23.30	0.92–0.93	2/2(100)	0.01–0.68	28/28(100)
Emotional functioning	4	0.87	62.69 ± 27.08	0.79–0.89	4/4(100)	0.03–0.71	56/56(100)
Cognitive functioning	2	0.69	73.48 ± 25.88	0.86–0.87	2/2(100)	0.01–0.63	28/28(100)
Social functioning	2	0.87	61.49 ± 28.62	0.93–0.94	2/2(100)	0.01–0.60	28/28(100)
Symptom scales/items							
Fatigue	3	0.67	32.32 ± 2.33	0.75–0.80	3/3(100)	0.05–0.65	42/42(100)
Nausea and vomiting	2	0.58	12.37 ± 18.72	0.005–0.10	0/2(0)	0.03–0.57	28/28(100)
Pain	2	0.57	27.15 ± 21.64	0.82–0.85	2/2(100)	0.22–0.63	28/28(100)
Dyspnoea	1	–	29.79 ± 31.97	1	1/1(100)	0.13–0.62	14/14(100)
Insomnia	1	–	26.26 ± 27.94	1	1/1(100)	0.06–0.61	14/14(100)
Appetite	1	–	25.00 ± 26.17	1	1/1(100)	0.003–0.51	14/14(100)
Constipation	1	–	27.27 ± 33.41	1	1/1(100)	0.03–0.32	14/14(100)
Diarrhoea	1	–	13.13 ± 23.57	1	1/1(100)	0.03–0.51	14/14(100)
Financial difficulties	1	–	57.83 ± 34.91	1	1/1(100)	0.19–0.62	14/14(100)

**Table 3:** Factor loadings^1^ of two construct solution

Domain	Factor 1	Factor 2
FACT-G		
Physical wellbeing	0.056	**0.714**
Social/family wellbeing	−0.024	**0.189**
Emotional wellbeing	0.011	**0.796**
Functional wellbeing	0.063	**0.517**
EORTC QLQ-30		
Physical functioning	**0.775**	0.006
Role functioning	**0.801**	−0.04
Emotional functioning	**0.794**	0.110
Cognitive functioning	**0.758**	−0.059
Social functioning	**0.776**	0.078

1. Extraction method: principal component with varimax rotation. The loading weights of the factor corresponding to the domains of each questionnaire are bolded

**Table 4:** Mean ± SD scores of the FACT-G and EORTC QLQ-C30 subscales based on taste and post-surgery radiation

Instruments	Taste				Post-surgery radiation				
	Increase	Decrease	Without change	p-value	Radio therapy	Chemotherapy	Both of them	None of them	p-value
FACT-G									
Physical wellbeing	20.50 ± 4.68	17.90 ± 4.75	16.21 ± 5.30	0.07	17.27 ± 5.52	17.11 ± 4.75	18.50 ± 4.89	16.19 ± 5.90	0.20
Social/family wellbeing	17.67 ± 5.59	16.46 ± 5.33	17.81 ± 4.90	0.33	18.64 ± 3.59	15.72 ± 5.71	16.33 ± 5.54	18.00 ± 4.78	0.17
Emotional wellbeing	12.83 ± 6.91	13.03 ± 4.18	13.05 ± 5.22	0.83	15.09 ± 4.04	12.00 ± 4.41	13.65 ± 4.67	12.46 ± 5.06	0.22
Functional wellbeing	16.50 ± 1.97	13.94 ± 3.90	15.35 ± 4.07	0.07	13.82 ± 3.06	14.94 ± 2.56	14.37 ± 3.88	15.12 ± 4.50	0.67
EORTC QLQ-C30									
Global health status/QOL	63.89 ± 16.39	53.97 ± 23.23	52.38 ± 21.97	0.48	50.76 ± 22.19	56.48 ± 20.72	52.17 ± 24.12	54.53 ± 21.83	0.86
Functional scales									
Physical functioning	82.22 ± 8.07	75.98 ± 17.04	73.86 ± 18.25	0.48	76.97 ± 9.12	77.41 ± 11.69	74.93 ± 18.92	74.50 ± 18.90	0.92
Role functioning	86.11 ± 16.39	76.19 ± 22.14	75.40 ± 25.02	0.56	71.21 ± 18.40	77.78 ± 22.14	76.45 ± 22.93	76.61 ± 25.17	0.89
Emotional functioning	73.61 ± 1.31	62.30 ± 27.59	62.04 ± 27.30	0.60	56.06 ± 19.75	61.57 ± 21.22	62.50 ± 31.9	64.47 ± 26.05	0.55
Cognitive functioning	83.33 ± 27.89	72.49 ± 25.43	73.55 ± 26.38	0.62	72.73 ± 28.16	69.44 ± 20.81	72.46 ± 26.11	75.73 ± 27.11	0.82
Social functioning	77.78 ± 22.77	61.90 ± 27.34	59.52 ± 30.19	0.33	62.12 ± 28.95	69.44 ± 26.97	58.33 ± 29.34	61.40 ± 28.72	0.59
Symptom scales/items									
Fatigue	24.07 ± 8.36	31.39 ± 21.54	34.04 ± 21.94	0.49	34.34 ± 16.62	28.40 ± 19.14	34.06 ± 24.16	31.77 ± 21.15	0.79
Nausea and vomiting	5.56 ± 13.61	14.02 ± 20.78	11.38 ± 16.89	0.49	6.06 ± 15.41	12.04 ± 20.47	14.86 ± 20.56	11.70 ± 17.24	0.55
Pain	13.89 ± 12.55	29.89 ± 20.77	25.66 ± 22.76	0.17	31.82 ± 11.68	24.07 ± 13.06	31.88 ± 24.55	23.39 ± 22.24	0.13
Dyspnoea	5.56 ± 13.61	30.69 ± 30.11	31.22 ± 34.33	0.16	27.27 ± 32.72	22.22 ± 22.87	34.06 ± 33.33	29.24 ± 33.38	0.59
Insomnia	33.33 ± 21.08	24.34 ± 28.84	27.51 ± 27.79	0.67	24.24 ± 21.56	18.52 ± 20.52	27.54 ± 31.67	28.07 ± 28.02	0.63
Appetite	22.22 ± 27.22	24.87 ± 25.38	25.40 ± 27.25	0.96	24.24 ± 30.15	18.52 ± 23.49	26.81 ± 23.95	25.73 ± 28.18	0.72
Constipation	5.56 ± 13.61	32.28 ± 35.40	24.34 ± 31.80	0.11	33.33 ± 39.44	35.19 ± 35.19	29.71 ± 33.87	21.64 ± 31.17	0.36
Diarrhoea	–	14.29 ± 25.20	13.23 ± 22.83	0.32	33.03 ± 10.05	12.96 ± 23.26	15.94 ± 26.05	12.87 ± 23.36	0.45
Financial difficulties	66.67 ± 21.08	58.20 ± 32.22	56.61 ± 38.63	0.84	57.58 ± 33.64	53.70 ± 34.56	63.04 ± 31.61	54.97 ± 38.05	0.65

## Discussion

There are a number of studies have compared the EORTC QLQ-C30 and FACT-G instruments [5]. However, these studies were limited to compare the EORTC QLQ-C30 and FACT-G with regard to content, reliability and validity, interpretability, availability of modules and accessibility of questionnaires. To our best knowledge, there is no study available to date to compare EORTC-C30 and FACT G simultaneously in a specific sample to prove whether EORTC-C30 and FACT G measure the same or different construct of quality of life. The results of the present research, along with the previous studies, offer guidance to assist clinicians in their choice of the two well-known generic HRQOL instruments that are commonly used in cancer clinical trials.

This study indicates that the Persian versions of EORTC QLQ-C30 and FACT-G are two reliable and valid instruments when applied to a sample of Iranian patients with total laryngectomy. Almost all domains in both instruments met the minimum internal consistency criterion of Cronbach’s alpha coefficient over 0.7, except for the “Functional wellbeing” subscale in FACT-G, and three symptoms subscales in the EORTC QLQ-C30. Moreover, our findings revealed that the instruments have excellent convergent and discriminant validity, except for nausea and vomiting symptom subscale in the EORTC QLQ-C30. This is in agreement with the findings of the previous studies on the psychometric properties of these questionnaires in patients with head-and-neck cancer in other languages and cultures [11–16, 18, 27, 30–32].

The two instruments have four subscales in common. The corresponding subscales in the FACT-G and EORTC QLQ-C30 are physical wellbeing versus physical functioning, emotional well-being versus emotional functioning, social/family wellbeing versus social functioning, functional wellbeing versus role functioning, respectively. However, the exploratory factor analysis extracted two different HRQOL factors: one corresponding to all of the domains of the EORTC QLQ-C30 and the other to all of the domains of the FACT-G, except for social functioning subscale which was not clearly loaded on the FACT-G measure. These findings indicate that the Persian versions of the EORTC QLQ-C30 and FACT-G measure two different constructs of HRQOL and neither can be used in place of the other. These findings are in agreement with those in the previous studies which found that the two instruments measure markedly different aspects of QOL in patients with breast cancer or Hodgkin’s disease, despite considerable overlap [33, 34]. However, the findings of the current study do not support the previous research reporting strong correlations between corresponding scales (physical, social, emotion, and role function) of these two instruments [35, 36].

There are several potential explanations for the observed discordant pattern among constructs of the EORTC QLQ-C30 and FACT-G. The inconsistency may be due to differences in goals adopted by questionnaire developers. The content and the form of the two instruments are different. While the FACT-G uses statements, the EORTC QLQ-C30 uses questions. Moreover, as compared with the FACT-G, items in the EORTC QLQ-C30 are more often negatively worded. It should be noted that negatively worded items can confuse respondents because of increasing difficulty in interpreting items, and consequently resulted in unsatisfactory item properties [37]. Even where the results justify the claim that the EORTC QLQ-C30 and FACT-G address similar issues [35], direct comparison of scores is not yet possible. This is because the two questionnaires differ in their item structure, response categories and scoring procedures [4]. Hence, Holzner et al. provided a practical guideline for converting scores from EORTC QLQ-C30 into the FACT-G and vice versa for use in oncological research [4].

Although in a previous research no significant patient preferences was observed for one of the two questionnaires, the EORTC QLQ-C30 was selected on the basis of its significantly better acceptability criteria [38]. Accordingly, if we intend to choose one of these questionnaires for measuring HRQOL in Iranian patients with total laryngectomy, EORTC QLQ-C30 would be the first choice. While the social function subscale in the FACT-G was not highly dependent to the FACT-G construct, all domains in the EORTC QLQ-C30 were highly correlated with their own construct. Moreover, as compared with FACT-G, EORTC QLQ-C30 has better internal consistency and discriminant validity. We also detected differences in sensitivity, precision and responsiveness between FACT-G and EORTC QLQ-C30. While in the FACT-G all items had ceiling effects less than 12%, more than half of the items in the EORTC QLQ-C30 had ceiling effects greater than 40%. The presence of ceiling effects in the EORTC QLQ-C30 indicates that the scale is less sensitive and efficient as compared to the FACT-G. This result was similar to a previous study that found ceiling effect was considerably larger for the EORTC QLQ-C30 compared to the FACT-G [4].

As demonstrated in previous quality of life studies, significant deterioration in taste functioning is among the most important complains in patients with total laryngectomy [3]. To this end, in the present study, we asked the patients to what extent their taste senses had changed after surgery. We found that there was no statistically significant difference in HRQOL scores between patients with different taste functioning. This finding indicates that EORTC QLQ-C30 and FACT-G are less sensitive and discriminative to differentiate between patients who have a poor taste functioning and those with a good taste functioning. The same result occurred for patients who had received different types of post-surgery radiation.

Our study has a number of limitations that need to be mentioned. The present study is a cross-sectional research and longitudinal study is needed to explore how patients’ QOL change over the course of treatment. Moreover, we specifically intended to assess interchangeability between the FACT-G and EORTC QLQ-C30 in patients with total laryngectomy. The findings revealed that the two instruments are not interchangeable. Although restricting the study sample to a homogenous group of patients with total laryngectomy can increase internal validity of the results, it may reduce external validity or generalizability of the findings to various subsites in head and neck cancer. With increasing internal validity due to a homogenous sample we are able to say that no other variables except the one we are studying caused the result. Hence, further studies with more focus on heterogeneous sample are suggested. Moreover, the study was conducted in a referral cancer surgery center in the south of Iran and included only patients with total laryngectomy, limiting the generalizability of the results. Finally, little is known about the comparative validity between generic and disease-specific instruments in patients with total laryngectomy. Ideally, the EORTC QLQ-C30 and FACT-G should be used with their own site specific modules including EORTC QLQ-H&N35 and FACT-H&N, respectively.

## Conclusions

The present study revealed that the EORTC QLQ-C30 and FACT-G measure distinct concepts related to HRQOL. Although four subscales in the two instruments may have similar titles, they measure different QOL issues. Accordingly, EORTC QLQ-C30 cannot be used as a substitute for the FACT-G with total laryngectomy patients. However, as compared to FACT-G, EORTC QLQ-C30 has advantages of producing specific symptom scores. In general, choosing an appropriate instrument depends on the nature of the individual study and the requirement for detailed specific information.

## Abbreviations

EORTC QLQ-30: European Organization for Research and Treatment of Cancer QoL Core Questionnaire;
FACT-G: Assessment of Cancer Therapy-General
HRQOL: Health related quality of life
QOL: Quality of life
TLM: Transoral laser microsurgery
